# Inguinal Endometriosis Visualized on I-131 Whole Body Scan

**DOI:** 10.4274/mirt.77044

**Published:** 2018-02-01

**Authors:** Derya Çayır, Mine Araz, Mahmut Apaydın, Erman Çakal

**Affiliations:** 1 University of Health Sciences, Dışkapı Yıldırım Beyazıt Training and Research Hospital, Clinic of Nuclear Medicine, Ankara, Turkey; 2 University of Health Sciences, Dışkapı Yıldırım Beyazıt Training and Research Hospital, Clinic of Endocrinology and Metabolism, Ankara, Turkey

**Keywords:** Endometriosis, whole body imaging, iodine-131

## Abstract

We present a rare case with inguinal iodine-131 (I-131) uptake on whole body scan. The patient was suffering from a painful right inguinal mass during menstrual period, which was later sonographically and histopathologically confirmed to be an inguinal focus of endometriosis. Endometriosis is a previously reported site of radioiodine uptake and detection of radioiodine uptake in the inguinal region has also been described. Nevertheless, to the best of our knowledge, this is the first case report of I-131 uptake in an inguinal endometriosis focus. History and physical examination of the patient are both very important in identifying the etiology of the ectopic uptake sites on I-131 whole body scan, and inguinal endometriosis should be kept in mind while reporting inguinal radioiodine uptake on I-131 whole body scan.

## Abstract (tr)

Bu raporda iyot-131 (I-131) tüm vücut taramada inguinal bölgede tutulum saptanan nadir bir olguyu sunuyoruz. Menstrüel siklus sırasında ağrılı inguinal kitle şikayeti olan hastada, kitlenin daha sonra sonografik ve histopatolojik olarak inguinal endometriozis odağına ait olduğu konfirme edildi. Endometrioziste radyoaktif iyot tutulumu ve inguinal bölgede radyoaktif iyot tutulumunun saptanması daha önce bildirilmiştir; ancak inguinal endometriozis odağında I-131 tutulumu, bildiğimiz kadarıyla, ilk kez bu olguda sunulmaktadır. I-131 tüm vücut taramada ektopik tutulum alanlarının etiyolojisinin çözülmesinde hasta öyküsü ve fizik muayene çok önemli olup, I-131 tüm vücut taramada inguinal bölgede radyoaktif iyot tutulumu raporlanırken inguinal endometriozis de akılda bulundurulmalıdır.

## Figures and Tables

**Figure 1:**
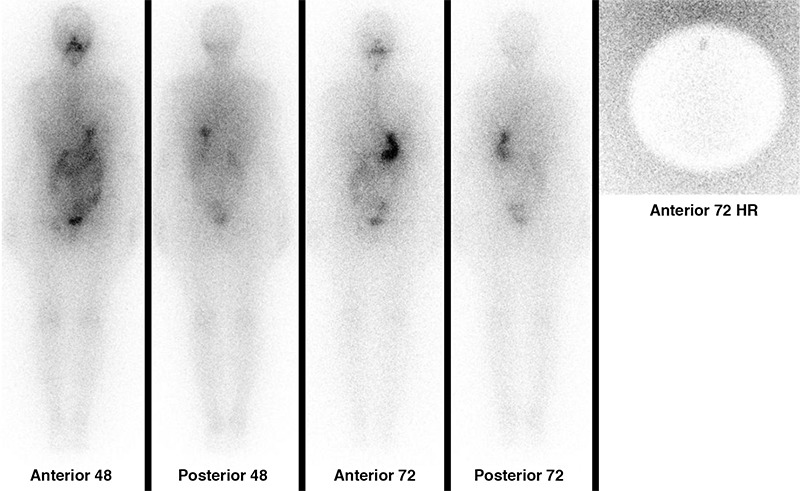
A 35-year-old woman with papillary thyroid carcinoma who had received 50 mCi iodine-131 (I-131) following total thyroidectomy underwent an I-131 whole body scan one year later. While serum levels of thyrotrophin-stimulating hormone was 92.22 mIU/L, thyroglobulin and anti-thyroglobulin antibody levels were 4.61 ng/mL and <0.9 IU/mL, respectively. I-131 whole body images were obtained by using a large field-of-view gamma camera (Siemens e.cam-signature; Siemens, Hoffmann Estates, Illinois, United States of America) in a 256x1024 matrix using a high energy parallel hole collimator and pinhole collimator at 48 and 72 hours. Images showed focal I-131 accumulation on the neck, located in the midline, along with I-131 uptake in the right inguinal region and the suprapubic area (Figure 1). The focal uptake on the neck was interpreted as residual thyroid tissue on the thyroglossal duct, and the suprapubic non-homogenous activity was attributed to uterine activity due to menstrual bleeding. The etiology of the right inguinal uptake was revealed with further evaluation. The patient was also suffering from groin pain during the menstrual period along with a right inguinal mass that enlarged at the same time. Right inguinal superficial ultrasound revealed a hypoechoic solid mass of 25x15 mm located in the neighborhood of femoral artery and multiple accompanying reactive lymph nodes with the largest dimensions measuring 43x8 mm. The Tru-Cut biopsy of the right inguinal palpable mass was performed that revealed endometriosis. Thyroglobulin elevation in our patient was associated with neck uptake and distant metastasis was excluded. Treatment was planned accordingly. Endometriosis is the existence of endometrium, the layer surrounding the uterine cavity, anywhere in the body other than the uterus. It is mostly located in the intrapelvic region (1). Inguinal (non-cutaneous) endometriosis is a rare presentation of endometriosis, occurring in only 0.6% of women (1,2). Inguinal endometriosis was first reported by Allen in 1896 (3,4,5). Patients with inguinal endometriosis complain of inguinal mass and pain, in particular, acute pain during menstrual cycles (2,6). The mechanism of iodine uptake in endometriosis is not yet clear, and iodine uptake in inguinal endometriosis has not been previously described in the literature. In our case, the scanning was performed during the patient’s menstrual cycle. An increase in blood flow to the inguinal endometriosis or presence of inflammation in this area might be the reasons for increased radioiodine uptake. Hyperemia, vasodilation, local edema, and increased capillary permeability may cause increased radioiodine uptake in inflamed areas (7,8). In our case, there were multiple reactive lymph nodes around the inguinal mass. Both increased blood flow and reactive lymph nodes could have led to an increased activity. In conclusion, although it is a well-established technique, one may still confront with unexpected findings on I-131 whole body scan. While reporting radioiodine uptake in the inguinal region, endometriosis should be kept in mind as a rare etiology and the patient should be further evaluated accordingly. Focal I-131 accumulation on the neck, located in the midline, is detected along with radioiodine uptake in the right inguinal region on I-131 whole body images.
